# Methylglyoxal-Mediated Stress Correlates with High Metabolic Activity and Promotes Tumor Growth in Colorectal Cancer

**DOI:** 10.3390/ijms18010213

**Published:** 2017-01-21

**Authors:** Barbara Chiavarina, Marie-Julie Nokin, Justine Bellier, Florence Durieux, Noëlla Bletard, Félicie Sherer, Pierre Lovinfosse, Olivier Peulen, Laurine Verset, Romain Dehon, Pieter Demetter, Andrei Turtoi, Koji Uchida, Serge Goldman, Roland Hustinx, Philippe Delvenne, Vincent Castronovo, Akeila Bellahcène

**Affiliations:** 1 Metastasis Research Laboratory, GIGA-Cancer, University of Liège, 4000 Liège, Belgium; barbarachiavarina@libero.it (B.C.); mjnokin@ulg.ac.be (M.-J.N.); Justine.Bellier@ulg.ac.be (J.B.); florence.durieux@student.ulg.ac.be (F.D.); Olivier.Peulen@ulg.ac.be (O.P.); A.Turtoi@ulg.ac.be (A.T.); vcastronovo@ulg.ac.be (V.C.); 2 Department of Pathology, Liège University Hospital, 4000 Liège, Belgium; nbletard@chu.ulg.ac.be (N.B.); P.Delvenne@ulg.ac.be (P.D.); 3 Department of Nuclear Medicine, Erasme University Hospital, Université Libre de Bruxelles, 1050 Bruxelles, Belgium; fsherer@ulb.ac.be (F.S.); sgoldman@ulb.ac.be (S.G.); 4 Nuclear Medicine and Oncological Imaging Division, Medical Physics Department, Liège University Hospital, 4000 Liège, Belgium; pierre.lovinfosse@chu.ulg.ac.be (P.L.); rhustinx@chu.ulg.ac.be (R.H.); 5 Department of Pathology, Erasme University Hospital, Université Libre de Bruxelles, 1050 Bruxelles, Belgium; Laurine.Verset@erasme.ulb.ac.be (L.V.); Romain.Dehon@erasme.ulb.ac.be (R.D.); Pieter.Demetter@erasme.ulb.ac.be (P.D.); 6 Laboratory of Food Chemistry, Department of Applied Biological Chemistry, Graduate School of Agricultural and Life Sciences, University of Tokyo, Tokyo 13-8654, Japan; a-uchida@mail.ecc.u-tokyo.ac.jp

**Keywords:** methylglyoxal, colorectal cancer, MG-adducts, glyoxalase 1, ^18^F-Fluorodeoxyglucose (18F-FDG)

## Abstract

Cancer cells generally rely on aerobic glycolysis as a major source of energy. Methylglyoxal (MG), a dicarbonyl compound that is produced as a side product during glycolysis, is highly reactive and induces the formation of advanced glycation end-products that are implicated in several pathologies including cancer. All mammalian cells have an enzymatic defense against MG composed by glyoxalases GLO1 and GLO2 that converts MG to d-lactate. Colorectal cancer (CRC) is one of the most frequently occurring cancers with high morbidity and mortality. In this study, we used immunohistochemistry to examine the level of MG protein adducts, in a series of 102 CRC human tumors divided into four clinical stages. We consistently detected a high level of MG adducts and low GLO1 activity in high stage tumors compared to low stage ones suggesting a pro-tumor role for dicarbonyl stress. Accordingly, GLO1 depletion in CRC cells promoted tumor growth in vivo that was efficiently reversed using carnosine, a potent MG scavenger. Our study represents the first demonstration that MG adducts accumulation is a consistent feature of high stage CRC tumors. Our data point to MG production and detoxification levels as an important molecular link between exacerbated glycolytic activity and CRC progression.

## 1. Introduction

Colorectal cancer (CRC) is the third most common cancer in the world and the fourth most common cause of cancer-related deaths [1,2]. Genomic instability and genetic alterations in tumor suppressor genes, including adenomatous polyposis coli (*APC*) and *p53*, and *oncogenes* like *K-Ras*, trigger the carcinogenesis process and consequently tumor progression [3,4,5]. Alterations in cellular metabolism are common events in cancer and specifically in colorectal cancer [6,7]. Indeed, cancer cells predominantly produce energy through glycolysis even in presence of oxygen, following the so-called Warburg effect [8,9,10]. Mutations in tumor-promoting and suppressor genes such as *Ras*, *c-Myc* and *p53* influence cell metabolism by notably inducing the overexpression of glucose transporters and glycolytic enzymes, thus leading to a phenotype which supports tumor growth and proliferation [11]. The increased glycolytic rate in tumor cells, make them accumulate high levels of reactive dicarbonyl compounds. Among those, methylglyoxal (MG), a highly reactive α-oxoaldehyde, is mainly generated in cells through the spontaneous degradation of triose phosphate intermediates of glycolysis, dihydroxyacetone phosphate and glyceraldehyde 3-phosphate [12]. Other minor sources of MG formation are the ketone body metabolism and the catabolism of threonine [13,14]. MG is a potent glycating agent, more reactive than glucose in glycation processes and able to modify proteins, lipids and nucleotides, generating dicarbonyl stress and cellular damage [15]. In all mammalian cells, MG is detoxified by the glyoxalase system, an enzymatic pathway consisting of two enzymes called glyoxalase 1 (GLO1) and glyoxalase 2 (GLO2) which catalyze the conversion of MG to d-lactate [16,17]. High GLO1 expression and activity levels have been described in many types of cancer including colon [18], prostate [19,20], lung [21], melanoma [22] and breast [23]. GLO1 amplification and overexpression have been correlated with cancer progression and drug resistance [24,25,26,27,28,29,30].

Increased glycolytic flux in cancer cells resulted in the development of ^18^F-FDG Positron emission tomography (PET) imaging of cancers to detect glucose uptake and assess tumor metabolism [31]. ^18^F-FDG, a glucose analog, is transported into the cell via glucose transporters but cannot proceed along the glycolytic pathway and is trapped within the cell. In CRC patients, imaging with ^18^F-FDG PET is considered as a useful tool to establish tumor stage before surgery and to detect tumor recurrence and metastases [32].

MG modifies arginine, lysine and cysteine residues in proteins and reacts with nucleic acids, thus generating advanced glycation end products (AGEs) among which hydroimidazolone (MG-H1) and argpyrimidine adducts are the more abundant [33]. Dicarbonyl stress has been well investigated in the context of diabetes where high levels of MG are linked with the pathogenesis of diabetic complications [34,35,36]. For example, arginine residues of arginine-glycine-aspartic acid (RGD) and glycine-phenylalanine-hydroxyproline-glycine-glutamate-arginine (GFOGER) domains of collagen IV are hot spots for MG glycation leading to decreased binding affinity and anoikis of endothelial cells [37]. Post-translational modification of voltage-gated sodium channel Na_v_1.8 by MG increased electrical excitability and facilitated firing of nociceptive neurons associated with hyperalgesia [38]. In summary, increased MG has been linked to nephropathy, retinopathy and neuropathy associated with diabetes [39,40].

To date the role of MG in cancer and the effects of dicarbonyl stress in tumorigenesis are still poorly investigated. One study reported the expression of argpyrimidine adducts in four different types of human cancers in a limited number of patients (5 cases per tumor type) [41]. We have recently described argpyrimidine adducts detection in a large series of human breast cancer lesions using an immunohistochemistry approach [42]. We reported for the first time the differential accumulation of argpyrimidine moieties among the four main breast cancer subtypes analyzed. We have shown that triple negative breast cancer tumors accumulated less argpyrimidine adducts than ER/PR positive, HER2 negative and HER2 positive lesions. In vitro, we demonstrated that triple negative breast cancer cells increased their level of GLO1 expression and activity in response to MG stress while ER/PR positive and HER2 positive cells remained stable under the same conditions. Remarkably, aggressive triple negative tumors were capable to exert a tight control on dicarbonyl stress by increasing their GLO1 detoxification capacity.

In cancer cells, the glycation of HSP27 by MG has been reported to inhibit Cyt *c*-mediated caspase activation. MG-modified HSP27 prevented cancer cell apoptosis in lung and gastrointestinal tumors and is associated to chemotherapy resistance [43,44]. During the preparation of this manuscript, a study provided evidence of the interaction between the components of the AGE-RAGE axis, GLO1 and adiponectin receptors in CRC, where these two latter proteins emerged as novel independent prognostic biomarkers of adverse significance for patients with early disease stage [45].

The present study aimed to investigate the importance of dicarbonyl stress in the context of CRC. Specifically we showed that high tumor MG adducts accumulation is associated with low GLO1 expression and activity levels and increased aggressiveness in colorectal cancer patients. In accordance with these observations, in vivo experiments using *GLO1*-depleted HCT116 colon cancer cells showed an increased tumor growth associated with MG adducts accumulation that can be reverted by carnosine, a potent MG scavenger.

To our knowledge, this is the first study demonstrating a correlation between dicarbonyl stress and CRC aggressiveness and to designate the blockade of dicarbonyl stress as a potential new therapeutic strategy for the treatment of CRC patients.

## 2. Results

### 2.1. Argpyrimidine Adducts Are Accumulated in CRC Cancer Tissues When Compared to Normal Counterpart

**Figure 1:**
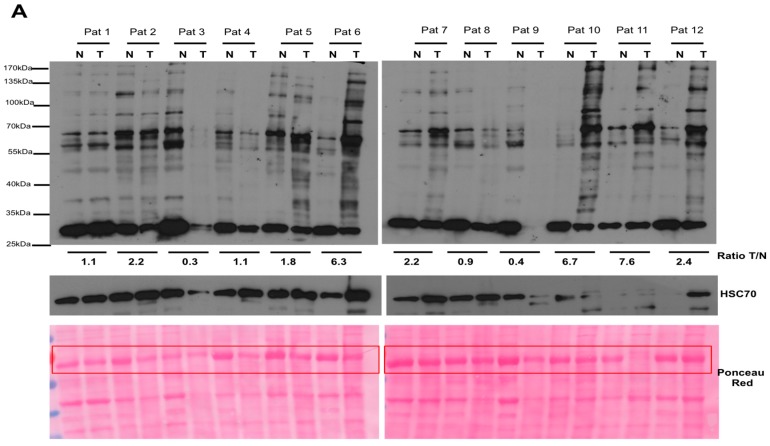

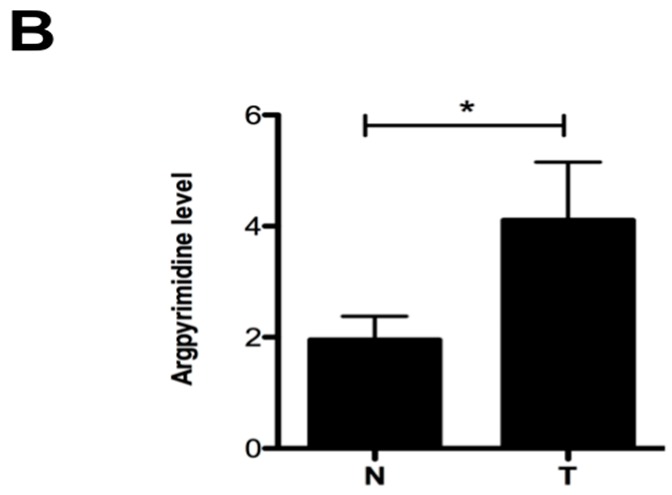
Argpyrimidine adducts are highly accumulated in colorectal cancer tissues when compared to matched non-cancer tissue. (**A**) Argpyrimidine adducts have been evaluated in 12 CRC tissues (T) and in their non-tumor counterpart (N) by Western blot analysis. HSC70 and Ponceau Red staining are both shown as loading control. The quantification of all visible bands corresponding to argpyrimidine adducts has been performed using ImageJ software (NIH Image, http://rsb.info.nih.gov/ij/, 1.42p, RRID:SCR_003070). Tumor to normal tissue (T/N) ratio of argpyrimidine adducts is shown for each patient. The most intense band of the Ponceau Red staining (boxed in red) has been used for the normalization; (**B**) Quantification of panel (A) data demonstrates a significant overall increase of MG-adducts in tumor tissues compared to normal counterpart. Bars represent the mean ± SEM of 12 patients analyzed. Statistical analysis has been performed using one way Anova, Mann–Whitney test and * *p* < 0.05.

Cancer cells use glycolysis as a major source of energy production. We evaluated the level of endogenous MG, a side product of glycolysis, in CRC using an antibody specifically directed against argpyrimidine adducts. The Western blot performed on 12 human CRC tissues and their normal counterparts is shown in Figure 1A. MG modified proteins are consistently more accumulated in tumor protein extracts than in the matched non tumor tissue (Figure 1B) indicating that MG-mediated dicarbonyl stress is more elevated in colon cancer cells than in normal tissue.

### 2.2. High Stage CRC Tumors Accumulate More Argpyrimidine Adducts When Compared with Low Stage Ones

**Figure 2:**
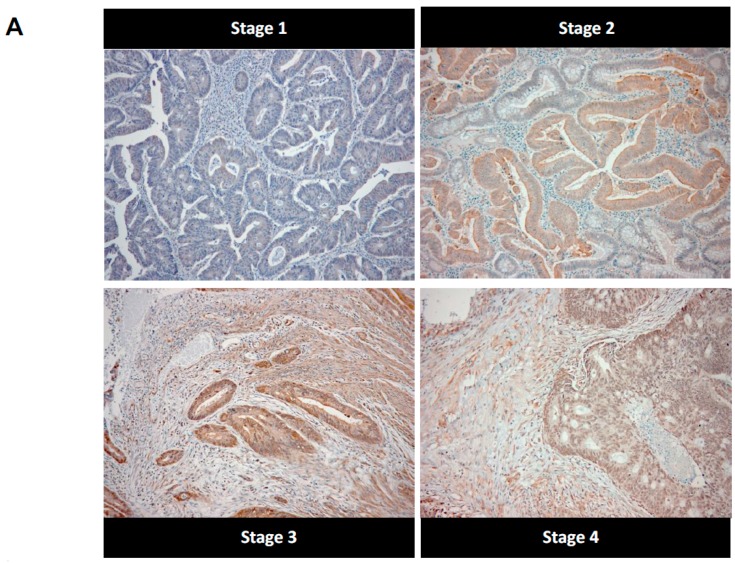

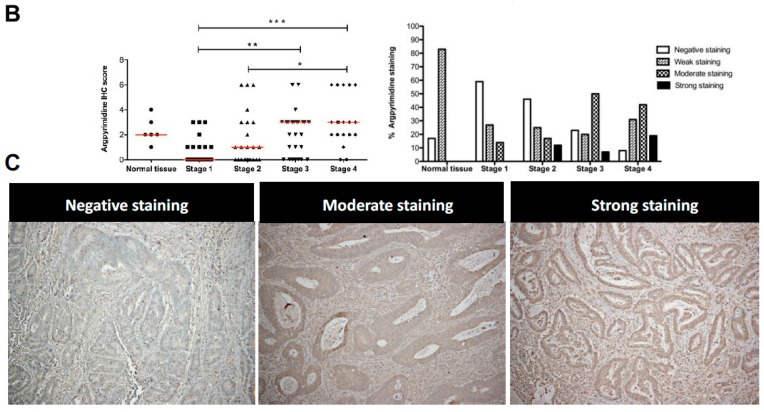

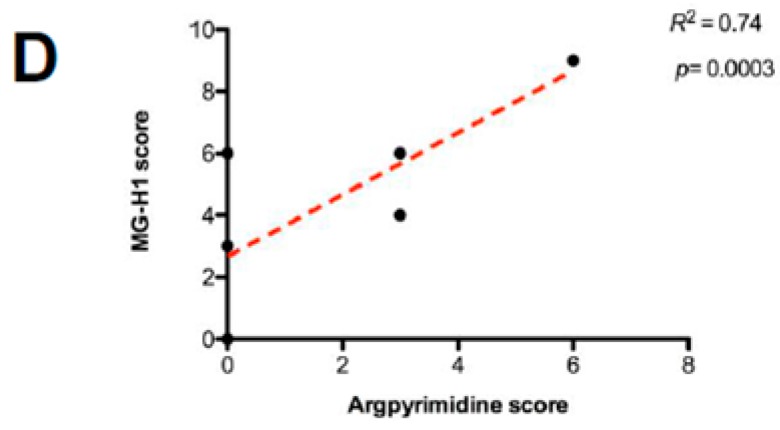
Consistent increase of argpyrimidine adducts in high stage tumors compared with low stage ones suggests a pro-tumor role for dicarbonyl stress. (**A**) Argpyrimidine adducts were examined in a series of 102 primary colorectal cancer patients samples grouped into four clinical stages (T1, T2, T3 and T4) and six normal colorectal tissues. One representative picture is shown for each stage analyzed (100× magnification); (**B**) Immunohistochemical quantification shows argpyrimidine staining evaluation divided into 4 groups (negative, weak, moderate and strong staining) based on the score values. Each dot represents one case and bars represent median. Statistical analysis has been performed using one-way ANOVA followed by Dunn’s Multiple Comparison Test and * *p* < 0.05, ** *p* < 0.01, *** *p* < 0.001. In the right panel, the percentage of negative, weak, moderate and strong argpyrimidine staining is shown for normal tissue and stage 1 to stage 4 tumors; (**C**) An IHC using an antibody against MG-H1 adducts has been performed on 12 CRC samples. IHC staining is shown for representative negative, moderate and strong staining (100× magnification). In accordance with argpyrimidine immunostaining, more MG-H1 adducts have been detected in the highest stage lesions; (**D**) Argpyrimidine and MG-H1 IHC detection showed a significant positive correlation (*R*^2^ = 0.74, *p* = 0.0003, Spearman rank correlation test).

We next examined the accumulation of argpyrimidine moieties on a collection of 102 colorectal cancer samples and six normal colon specimens using immunohistochemistry. Argpyrimidine staining was mainly localized in the cytoplasm, however in some tumor samples a nuclear staining was clearly detectable. In good accordance with the Western blot analysis conducted on non-tumor colon samples, a negative to weak argpyrimidine staining was observed in the normal colon tissue samples (Figure 2B). We divided the cohort of patients into four main groups, based on the histological features, from stage 1 to stage 4. As shown in Figure 2A,B, argpyrimidine staining was strongly detectable in high stage tumors and only weakly in the low stage ones suggesting a pro-tumor role of dicarbonyl stress in CRC. We confirmed this observation using an antibody directed against MG-H1 adducts (Figure 2C) on a representative number of tumors (3 of each stage). In good accordance with argpyrimidine immunostaining, we detected more MG-H1 adducts in the high stage lesions. We demonstrated a significant positive correlation (*R*^2^ = 0.74, *p* = 0.0003) between argpyrimidine and MG-H1 scores (Figure 2D) thus reinforcing the signature of MG-mediated dicarbonyl stress in CRC tumors.

### 2.3. Highly Glycolytic CRC Tumors Present with High Dicarbonyl Stress

**Figure 3:**
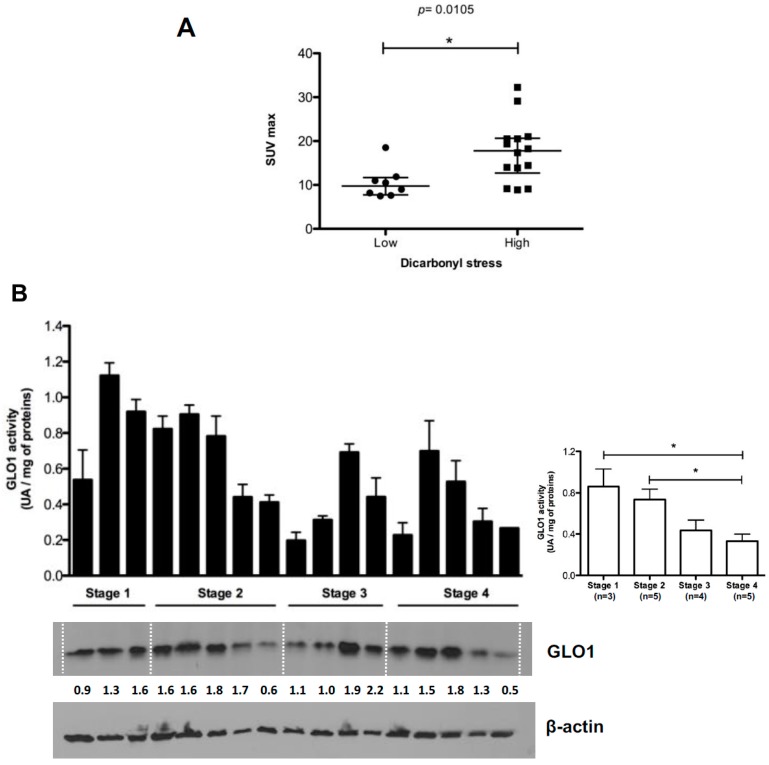

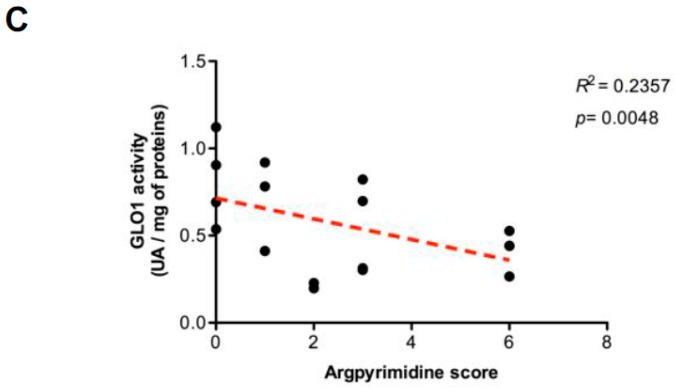
^18^F-FDG PET activity and argpyrimidine accumulation in CRC patients. (**A**) SUVmax is significantly higher in tumors with high dicarbonyl stress. Each dot represents one case and bars represent mean ± SEM. (*p* = 0.0105, Mann–Whitney test); (**B**) GLO1 activity and expression were evaluated in stage 1 (*n* = 3), stage 2 (*n* = 5), stage 3 (*n* = 4) and stage 4 (*n* = 5) colorectal cancer patients. **β-**Actin is used as loading control. Right panel, GLO1 activity is significantly higher in low stage tumors compared with high stage ones. Average of three technical replicates ± SEM is shown (Neuwman–Keul Tests, * *p* < 0.05); (**C**) The correlation analysis performed between argpyrimidine immunostaining score and GLO1 activity evaluated on 17 CRC patients demonstrated a significant inverse correlation between these two parameters (Spearman rank correlation test).

Cancer cells are different from normal tissues as they take up glucose at a high rate to sustain their boosted aerobic glycolysis. Accumulation of ^18^F-FDG in tumors is based on enhanced glucose metabolism and has been shown to correlate with tumor growth rate. To evaluate the potential relationship between ^18^F-FDG accumulation and dicarbonyl stress in CRC patients, we analyzed available PET data from 25 CRC patients which tumors were grouped according to their argpyrimidine immunohistochemistry (IHC) scores into low (score from 0 to 2) and high (score from 3 to 9) dicarbonyl stress. The analysis of the metabolic activity was based on the measure of maximal standardized uptake value (SUVmax), mean standardized uptake value (SUVmean) and total lesion glycolysis (TLG) that are commonly used to assess malignant tumor metabolism. We observed that SUVmax was significantly higher in tumors with high dicarbonyl stress than in low dicarbonyl stress lesions indicating a positive association between dicarbonyl stress and high metabolic activity in CRC tumors (Figure 3A). SUVmean and TLG values did not correlate significantly with argpyrimidine staining (data not shown). As shown in Figure 3B, GLO1 activity and expression measured on protein CRC tumor extracts are lower in high stage tumors compared with low stage ones. Moreover, GLO1 activity and argpyrimidine staining evaluated on the same CRC tumor samples were inversely correlated (Figure 3C). These results are in good accordance with the significant accumulation of argpyrimidine adducts observed in invasive tumors when compared with less aggressive tumors. Finally, we evaluated the possibility of a correlation between argpyrimidine immunostaining and lymph nodes involvement in 92 CRC patients but we did not observe any significant relationship between the two parameters suggesting that dicarbonyl stress is not associated with lymph nodes invasion in CRC (data not shown).

### 2.4. Evaluation of MG-Adducts, GLO1 Expression and Activity in Human CRC Cell Lines

**Figure 4:**
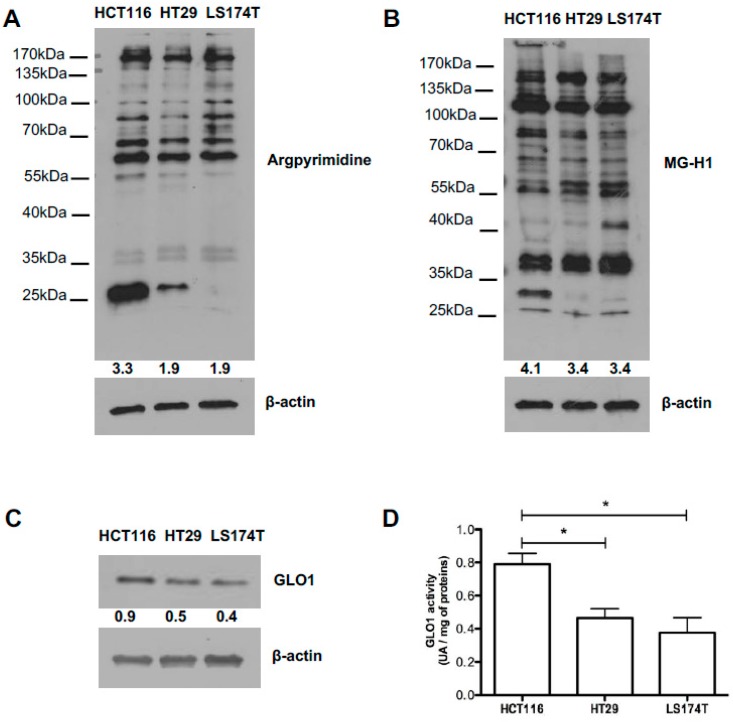
In vitro characterization of colorectal cancer cell lines. (**A**) Argpyrimidine and (**B**) MG-H1 accumulation has been evaluated in HCT116, HT29 and LS174T human colorectal cancer cell lines using Western blot analysis; (**C**) GLO1 basal expression and (**D**) activity in HCT116, HT29 and LS174T cells. All immunoblots were quantified by densitometric analysis and normalized for β-actin. Statistical analysis has been performed using Newman–Keuls multiple comparison test, * *p* < 0.05. All data are representative of three independent experiments (*n* = 3) as the mean values ± SEM.

Next, we investigated argpyrimidine and MG-H1 accumulation in HCT116, HT29 and LS174T human CRC cell lines using Western blot analysis. In all the cell lines evaluated, we detected a basal accumulation of MG-modified proteins, in particular HCT116 cells showed more adducts compared with the two other cell lines (Figure 4A,B). The three cell lines expressed GLO1 at protein level however the activity was significantly higher in HCT116 cells when compared with HT29 and LS174T cells (Figure 4C,D).

### 2.5. GLO1 Depletion Favors Colorectal Cancer Cell Growth In Vivo: An Effect That Is Reversed by Carnosine

**Figure 5:**
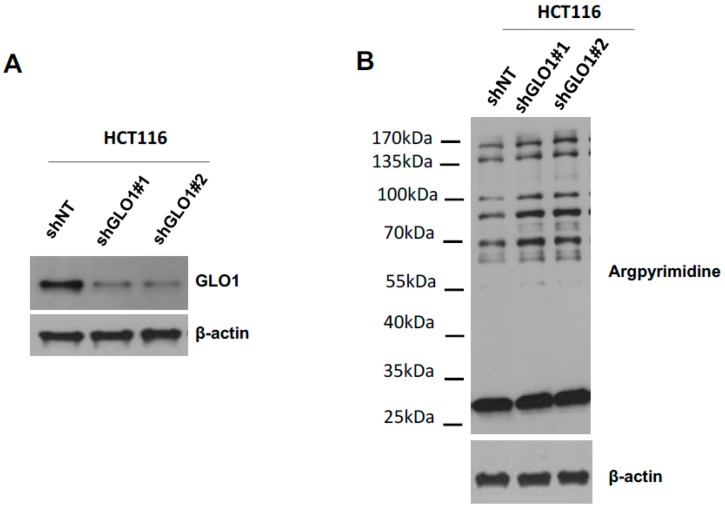

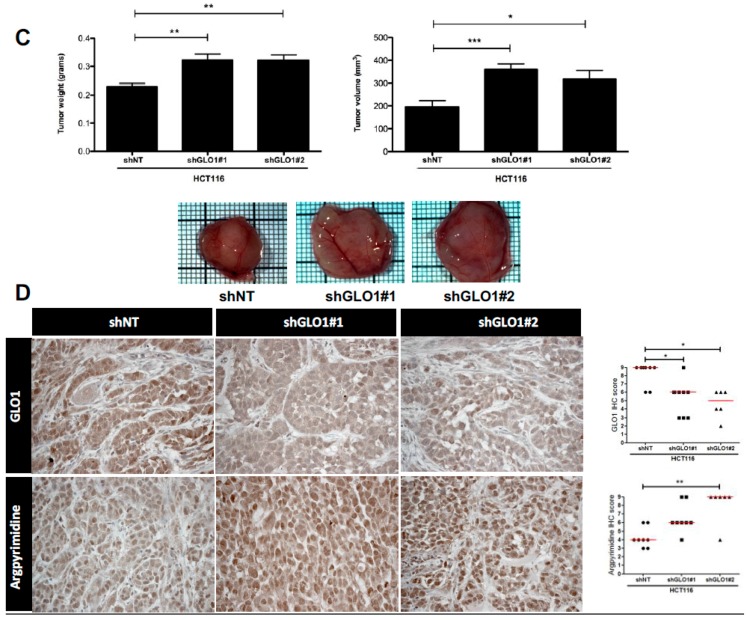
*GLO1* depletion favors colorectal cancer cell growth in vivo. (**A**) The efficiency of *GLO1* knockdown in HCT116 cells was validated by Western blot analysis; (**B**) Increased argpyrimidine level has been observed in *GLO1* depleted cells compared with shNT control cells using Western blot analysis. **β**-Actin is shown as loading control; (**C**) Effect of *GLO1* silencing on HCT116-derived tumor growth in chorioallantoic membrane (CAM) tumor model. The weight (**left**) and volume (**right**) of CAM experimental tumors collected at Day 7 is shown (at least 6 eggs/group). Data are shown as mean values ± SEM. Representative macroscopic tumor appearance is shown for each condition according to CAM experiment details are described in Material and Methods; (**D**) Representative GLO1 expression and argpyrimidine levels in CAM experimental tumors (400× magnification). IHC scoring for each is shown in panels on the right. Each dot represents one case and bars represent median. Statistical analysis has been performed using Bonferroni Multiple Comparison Test, * *p* < 0.05, ** *p* < 0.01, *** *p* < 0.001.

To evaluate whether the loss of GLO1 in CRC cells had an impact on tumor growth in vivo, we set-up a silencing strategy in HCT116 cells. We used two shRNAs specifically directed against *GLO1* as a model to stably induce high endogenous MG stress in HCT116 cells. The efficiency of *GLO1* silencing has been validated at the protein level and an increased MG-adducts level has been evidenced in stably depleted clones (Figure 5A,B). Next, we implanted stably GLO1 depleted HCT116 cells on the chick embryo chorioallantoic membrane (CAM) and evaluated tumor development seven days post-implantation. As shown in Figure 5C, *GLO1* depleted cells showed an increase in tumor growth compared to control cells. Histologic analysis performed on sections of *GLO1* depleted tumors validated GLO1 expression down regulation (Figure 5D) and showed higher accumulation of MG-adducts when compared with control tumors (Figure 5D).

**Figure 6:**
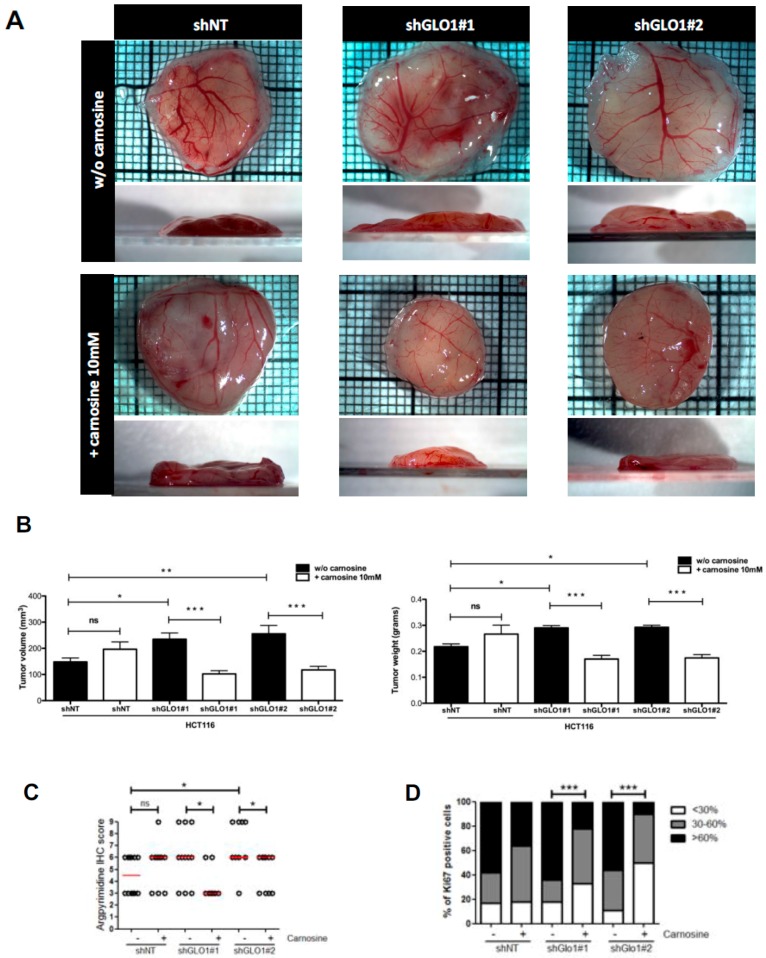
Carnosine treatment of *GLO1*-depleted HCT116 cells inhibits tumor growth in vivo. HCT116 shGLO1#1 and #2 and shNT control cells were implanted on chorioallantoic (CAM) tumor model. Cancer cells were treated with carnosine (10 mM) from the day after implantation on CAM until the end of the experiment. Tumor growth has been evaluated seven days post-implantation (at least 10 eggs/group) as described under Material and Methods section. (**A**) Representative macroscopic tumor appearance in each condition is shown; (**B**) Reduction of tumor volume (**left** **panel**) and weight (**right** **panel**) after carnosine treatment of *GLO1* depleted HCT116-derived tumors, data are shown as mean values ± SEM. Statistical analysis has been performed using Bonferroni Multiple Comparison Test; (**C**) Significant decrease of argpyrimidine level in experimental CAM tumors upon carnosine treatment. Each dot represents one case and bars represent median. Statistical analysis has been performed using Dunn’s Multiple Comparison Test and Mann–Whitney test; (**D**) Percentage of Ki67 positive cells in experimental CAM tumors upon carnosine treatment. Statistical analysis has been performed using Chi-square Contingency Test. * *p* < 0.05, ** *p* < 0.01, *** *p* < 0.001, and ns = not significant.

Finally, to assess the direct involvement of MG stress on the growth of *GLO1* depleted HCT116 cells, we treated engrafted cancer cells with carnosine (10 mM), a MG scavenger, from the day after implantation on CAM until the end of the experiment as described under Material and Methods section. We observed that tumor growth and volume were almost twofold decreased in sh*GLO1* tumors treated with carnosine compared with the non-treated ones. Interestingly, carnosine treatment did not affect the growth of HCT116 control tumors (Figure 6A,B). IHC analysis performed on collected CAM tumors revealed the effective reduction of argpyrimidine adducts in sh*GLO1* tumors treated with carnosine (Figure 6C). A significant decrease of the proportion of Ki67 positive cells in sh*GLO1* tumors under carnosine treatment sustained the reduction of tumor growth (Figure 6D). Our data indicate that the pro-tumor effect of *GLO1* silencing in CRC cells is linked with MG stress and can be blocked using a MG scavenger.

## 3. Discussion

One well-established metabolic abnormality in cancer cells is the Warburg effect, which demonstrates an increased glycolysis even in the presence of oxygen. Consequently, tumor cells inevitably accumulate MG, which will affect both their proteome and genome. Through its specific interaction with proteins, MG notably induces the formation of argpyrimidine and MG-H1 adducts. MG stress and the glyoxalase detoxification system have been well studied in relation with diabetes and its complications [46]. CRC is one of the most common cancers worldwide, with the highest incidence rates in Western countries. Hyperglycemia associated with diabetes is a well-established risk factor of colon cancer and altered glucose metabolism has been recently associated with the development of colorectal adenomas, the pathological precursors of CRC [47]. In this study, we showed that CRC tumors accumulate significantly more MG adducts than normal colon, which is consistent with the predicted higher glucose metabolic rates in cancer than in normal cells. Interestingly, we observed that primary tumor staging positively correlated with MG adducts detection indicating for the first time that CRC tumor aggressiveness, evaluated here in term of how deeply the primary tumor has grown into the bowel lining, is associated with the degree of dicarbonyl stress. We found a consistent decrease of GLO1 expression and enzymatic activity in high stage when compared with low stage tumors which could explain, at least in part, their increased level of MG adducts.

*GLO1* gene has been reported to be overexpressed and/or amplified in several types of cancer and has been thus considered as a novel oncogene which suppression using specific inhibitors or gene silencing strategies could abolish tumor growth via toxic MG accumulation [24,25,26,27,28,48]. However, others and we recently established that *GLO1* must also be considered as a tumor suppressor. In their study aimed at functionally identifying tumor suppressor genes in liver cancer, Zender and collaborators identified *GLO1* gene which knockdown using specific shRNAs increased tumor growth in a mouse model [49]. In our hands, the use of a stably depleted *GLO1* xenograft tumor model in vivo allowed the demonstration of the pro-tumorigenic and pro-metastatic role of endogenous MG accumulation in breast cancer cells [50]. In line with our previous study, we show here that *GLO1* depletion in colon cancer cells induces an increased level of argpyrimidine adducts and enhanced tumor growth in vivo. The reversion of dicarbonyl stress onset using carnosine, a well described MG scavenger [51], impeded tumor development indicating that pro-tumorigenic effects associated with *GLO1* inhibition were dependent upon MG glycating activity.

In summary, the ambivalent role demonstrated for *GLO1* as a tumor promoter or suppressor is likely to be cancer type-dependent and it is expectable that cell lines with dissimilar backgrounds and MG detoxification rates will respond differently to MG stress. For example, several studies have reported that cultured cancer cells presenting with *GLO1* gene amplification are more sensitive to *GLO1* inhibition, in terms of growth inhibition and/or apoptosis induction, than cancer cells with normal *GLO1* gene copy number (21,24). These data indicate that only a subset of cancer cells is highly dependent on GLO1 activity to grow and survive under high MG stress condition. In cancer cells, a better characterization of the main factors that control the level of dependence on GLO1 is needed before GLO1 inhibitors could be envisaged as promising anti-cancer drugs.

In the clinic, the avidity of CRC tumor cells for glucose is used for monitoring treatment response and the detection of recurrence using ^18^F-FDG PET imaging [32]. High SUVmax is associated with bad outcome and is considered as a marker of poor prognosis in CRC [52]. On a subset of CRC tumors, we have been able to demonstrate a significant positive association between SUVmax values and dicarbonyl stress as assessed by argpyrimidine level in tumors. Our small-scale analysis demonstrates the practical utility of argpyrimidine detection using IHC in CRC tumors as a read out of their elevated glycolytic metabolism. Studies on larger series of samples are needed to conclusively document the relationship between argpyrimidine detection and tumor metabolic status in CRC patients.

Considering its cytotoxic effect, MG has been first proposed as an anti-cancer agent in human leukemia [53]. However, several studies have demonstrated a pro-cancer role for MG, notably through its glycation of heat shock protein HSP27, which supports the escape of cancer cells from apoptosis [43,44,54]. We have recently reported in breast cancer that HSP90 is also a target of MG and mediates its pro-cancer role [50]. We have shown that post-translational glycation of HSP90 affects its activity and decreases the level of large tumor suppressor 1 (LATS1), a key kinase regulating the Hippo pathway through Yes-associated protein (YAP). Under MG stress, YAP is sequestrated in the nucleus where it positively regulates target genes known to promote cell growth and proliferation. Using cancer cells stably depleted for *GLO1* as a model of high endogenous MG, we demonstrated in vivo the pro-tumorigenic and pro-metastatic roles of dicarbonyl stress in breast cancer.

This study adds new evidence to sustain a major role of non-enzymatic glycation in the regulation of tumor growth. The administration of a MG scavenger in combination with conventional chemotherapy could represent a good strategy of treatment for patients presenting with aggressive CRC. Previous studies reported the anti-tumorigenic effect of carnosine in glioblastoma, gastric cancer and CRC [55,56,57]. These reports essentially focused on carnosine effect on cell proliferation. Ongoing studies in our laboratory will help identify specific protein targets of MG in cancer cells and explore further the molecular mechanisms of action of carnosine as a blocker of MG-mediated dicarbonyl stress in highly glycolytic tumors.

It is well established that type 2 diabetes and cancer have several common risk factors such as obesity, sex, aging and diet. In addition, in diabetic patients, insulin resistance and hyperinsulinemia are two independent risk factors for cancer development [58,59]. Diabetic patients present higher levels of circulating MG leading to formation of MG adducts and consequently to several diabetes clinical complications like vascular damage, nephropathy and inflammation [60]. Several in vitro and in vivo studies reported that metformin, the drug of choice for type 2 diabetes management, inhibits cancer cell growth [61]. Zhang and collaborators have concluded from their meta-analysis of five observational studies that metformin appeared to be associated with reduced risk of CRC incidence in diabetic patients in comparison with other hypoglycemic drugs [62]. In the light of our data, it is tempting to speculate that anti-carcinogenic effects of metformin could also be attributed, at least in part, to its potent MG scavenging activity [61]. Future studies should evaluate the extent to which metformin interferes with dicarbonyl stress and glyoxalase system in highly glycolytic cancer cells. Most importantly, our study reinforces the critical link between dicarbonyl stress and cancer and designates energetic metabolism switch in CRC as a target for future innovative therapeutic strategies.

## 4. Materials and Methods

### 4.1. Clinical Tumor Samples

The immunohistochemical analysis has been performed on a total number of 102 CRC tumors and 6 normal colon biopsies obtained from patients in whom no abnormalities of colonic mucosa were detected during colonoscopy. Sex, age and pTNM status were retrieved from medical reports and are summarized in Table 1. In the current study, the use of human material has been approved by the ethical committee of the University of Liège (ethics committee approval number 2013/302). ^18^FDG-PET scans data (SUVmax, SUVmean and TLG values) were available for 11 CRC patients from the University Hospital of Liège and from 14 supplementary patients from Erasme University Hospital, Brussels, Belgium (ethics committee approval number P2009/264). Western blot analysis was conducted on total protein extracts from an extra series of 12 colon adenocarcinoma (stage 2, 3 or 4 from University Hospital of Liège) and their matched non tumor counterpart. All human tissue samples represent residual histological material that was obtained from the indicated hospitals in good accordance with the institutional ethical guidelines.

### 4.2. Cell Lines

Colorectal cancer cell lines HCT116 and LS174T were obtained from the American Type Culture Collection (ATCC). HT29 cells were a kind gift from Dr. P. Close (University of Liège, Belgium). All cell lines were cultured in DMEM (standard glucose concentration of 4.5 g/L, Lonza, Basel, Switzerland) containing 10% fetal bovine serum and 2 mM l-glutamine.

### 4.3. shRNA Transfection

HCT116 cells were stably transfected with sh*GLO1*#1 and sh*GLO1*#2 and shNT. *GLO1*-shRNAs plasmids and non-target shRNA plasmid were provided from Sigma-Aldrich, TRCN0000118627 (#1), TRCN0000118628 (#2), SHC005 (shNT). HCT116 transfected cells were selected with puromycin (0.5 μg/mL, Sigma-Aldrich, Saint-Louis, MO, USA).

### 4.4. Western Blot Analysis and Antibodies

Cell and tissue samples were extracted in RIPA buffer (150 mM NaCl, 0.5% Na-deoxycholate, 1% Triton X-100, 0.1%SDS, 50 mM Tris-HCl 1 M pH 7.5) containing protease and phosphatase inhibitors (Roche, Mannheim, Germany). After incubation under rotation at 4 °C for 40 min, lysates were then centrifugated at 14,000× *g* for 15 min at 4 °C to remove insoluble debris. Protein concentrations were determined using the BCA assay (Pierce, Rockford, IL, USA). Twenty μg of proteins were then separated by Sodium dodecyl sulfate polyacrylamide gel electrophoresis (SDS-PAGE) and transferred to a PVDF membrane. After, the membranes were blocked in TBS-Tween 0.1% containing 5% nonfat dried milk (Bio-Rad, Hercules, CA, USA) and incubated with the selected primary antibodies overnight at 4 °C. The membranes were probed with anti-argpyrimidine (mAb6B) monoclonal antibody (1:6000). The specificity of the argpyrimidine antibody has been previously demonstrated using competitive ELISA and it has been shown to not react with other MG-arginine adducts such as 5-hydro-5-methylimidazolone and tetrahydropyrimidine [63]. Other sources of antibodies were as follows: anti-GLO1 monoclonal antibody (1:1000 dilution, cat#02-14, BioMac, Leipzig, Germany); anti-beta-actin (1:5000 dilution, cat# A5441, Sigma, Saint-Louis, MO, USA); anti-HSC70 (1:5000, cat# sc7298, Santa-Cruz, Dallas, TX, USA). Horseradish peroxidase-conjugated secondary antibodies (anti-mouse, 1:6000 dilution (Dako, Carpinteria, CA, USA) or anti-rabbit 1:3000 (Invitrogen, Carlsbad, CA, USA) were used to visualize bound primary antibodies, with the Enhanced Chemiluminescence (ECL) Western blotting substrate (Pierce).

### 4.5. Immunohistochemistry

Formalin-fixed paraffin-embedded sections of patients material and experimental CAM tumors were deparaffinized in xylene and rehydrated. To block endogenous peroxidase activity, the slides were treated with 3% hydrogen peroxide in methanol for 30 min and washed in PBS for 20 min. Antigen retrieval was performed in 10 mM sodium citrate, pH 6 for 40 min at 95 °C. Sections were then incubated with 1.5% normal horse serum (cat#S-2000, Vector Laboratories, Burlingame, CA, USA) for 30 min to block the nonspecific serum-binding sites. Then, sections were incubated overnight at 4 °C with anti-argpyrimidine (1:1000 dilution), anti-MG-H1 (1:50 dilution, 3D11 clone Cell Biolabs, Inc., San Diego, CA, USA), anti-human GLO1 (1:100 dilution) and anti-Ki67 (1:100 dilution) antibodies. Antibody binding was detected using an anti-mouse biotinylated secondary (cat#BA-2000, Vector Laboratories) for 30 min followed by incubation with the avidin-biotin-peroxidase complex (Vectastain ABC Kit, Vector Laboratories). Immunoreactivity was revealed using 3,3′-diaminobenzidine tetrahydrochloride (DAB). The slides were counterstained with hematoxylin, dehydrated and mounted.

### 4.6. Evaluation of Immunohistochemical Staining

The immunohistochemically stained sections were analyzed and scored by an anatomopathologist (N.B). IHC evaluation has been performed according to the intensity of the staining (0, 1+, 2+, 3+) and the percentage of positive cancer cells (0%–25% = 0, 25%–50% = 1, 50%–75% = 2, 75%–100% = 3). As we described previously [64], the results obtained with the 2 scales were multiplied together yielding a single scale with steps of 0, 1+, 2+, 3+, 4+, 6+ and 9+ where 0, 1+ and 2+ scores were considered to be negative or weak staining and 3+, 4+, 6+ and 9+ scores were considered to be medium or strong staining.

### 4.7. GLO1 Activity Assay

The basal activity of GLO1 was measured in colorectal cancer cell lines and in 17 frozen CRC tissue samples after protein extraction in RIPA buffer by measuring the *S*-d-lactoylglutathione formation from the hemimercaptal obtained by preincubation of an equimolar (1 mM) mixture of MG (cat#M0252, Sigma) and GSH (cat#G4251, Sigma) in 50 mM sodium phosphate buffer, pH 6.8, at 25 °C for 15 min. Then, the reaction mixture was added to 20 µg of tumoral or cell line extracts in a final volume of 210 µL. *S*-d-lactoylglutathione formation was followed spectrophotometrically by the increase in absorbance at 240 nm at 25 °C. GLO1 activity is defined as arbitrary units (A.U.) of enzyme per mg of proteins.

### 4.8. Chicken Chorioallantoic Membrane In Vivo Tumor Assay

On Embryonic Day 11, 100 μL of a suspension of 2 × 10^6^ of HCT116 shNT, sh*GLO1*#1 and sh*GLO1*#2 cells in culture medium mixed (1:1) with Matrigel (BD Biosciences) were deposited in the center of a plastic ring on the chorioallantoic membrane (CAM). Carnosine (cat#C9625, Sigma) treatment at a concentration of 10 mM in saline solution was performed daily from the day after cell implantation until the end of the experiment. Control eggs received saline solution following the same schedule, at least 6 eggs were used per condition. Tumors were harvested on Embryonic Day 18 and were fixed in 4% paraformaldehyde solution (30 min) for histology analysis. Tumor volume was measured using the formula *V* = 4/3 π × *H/2* × *L/2* × *W/2* where *H*, *L*, and *W* denote height, length, and width of the tumor, respectively.

### 4.9. Statistical Analysis

The data were statistically analyzed using either two-tailed Student t-test or with one-way ANOVA followed by specific multiple comparison tests as indicated in figure legends. *p*-values less than 0.05 were considered statistically significant.

## Figures and Tables

**Table 1:** Characteristics of CRC patients cohort (*n* = 102).

Characteristics	*n*	%
Age, years		
Median	72	
Range	25–92	
Histology		
pTNM		
T1	22	21.6
T2	24	23.5
T3	30	29.4
T4	26	25.5
N0	56	54.9
N1	25	24.5
N2	11	10.8
Nx	10	9.8
M0	0	0
M1	6	5.9
Mx	96	94.1

pTNM indicates the post-chirurgical histopathological classification; Nx and Mx: lymph nodes and distant metastases cannot be evaluated, respectively.
